# Risk factors for cervical cancer in Morocco: a case-control study

**DOI:** 10.11604/pamj.2025.50.87.45024

**Published:** 2025-03-28

**Authors:** Malika Allali, Khaoula Errafii, Rachid El Fermi, Nouha Messaoudi, Karima Fichtali, Hicham El Fazazi, Adil El Ghanmi, Sanaa El Majjaoui, Nabil Ismaili, Lahcen Wakrim, Najib Al Idrissi, Abdelaziz Wajih Rhalem, Bouchra Ghazi, Ahd Ouladlahsen, Youssef Bakri, Hassan Ghazal, Salsabil Hamdi

**Affiliations:** 1 Virology and Public Health Laboratory, *Centre de Serums et Vaccins (Institut Pasteur du Maroc)*, Casablanca, Morocco,; 2 Laboratory of Human Pathologies Biology, Department of Biology, Faculty of Sciences and Genomic Center of Human Pathologies, Faculty of Medicine and Pharmacy, University Mohammed V, Rabat, Morocco,; 3 African Genome Center, Mohamed IV Polytechnic University, Benguerir 43151, Morocco,; 4 Hospital Cheikh Khalifa Ibn Zaid, Casablanca, Morocco,; 5 Fertility Center Cheikh Zaid International University Hospital, Abulcasis International University of Health Sciences, Rabat, Morocco,; 6 Université Mohammed VI des Sciences et de la Santé, Casablanca, Maroc,; 7 Laboratoire de Génomique, Epigénétique, Médecine Personnalisée et Prédictive, Université Mohammed VI des Sciences et de la Santé, Casablanca, Maroc,; 8 Research Team E2SN, ENSAM, Mohammed V University in Rabat, Rabat, Morocco,; 9 Department of Infectious Diseases, Hassan II University, Casablanca, Morocco,; 10 Scientific Department, National Center for Scientific and Technical Research, Rabat, Morocco

**Keywords:** Cervical cancer, risk factors, prevalence, human papillomavirus, phylogenetic, Morocco

## Abstract

**Introduction:**

Cervical cancer (CC) is one of the most common malignancies among women in Morocco. This study aims to evaluate the risk factors associated with CC in Moroccan women aged 18 to 62.

**Methods:**

this was a case-control study of 169 women who received radio-chemotherapy and 100 controls. Statistical analyses were performed using SPSS version 29.0.10 to determine associated factors at a significance level of ≤0.05.

**Results:**

significant associations were found between CC and the following factors: educational level (OR= 9.167), sexual activity during menstruation (OR= 2.351), previous occurrences of sexually transmitted infections (OR= 2.173), and history of multiple sexual partners by the husband (OR= 6.305). However, family history of cancer, consanguinity, number of sleeping hours, and stress did not show any significant association with CC. HPV infection was detected in 33.81% of cases and 8% of controls, with HPV16 being the most prevalent genotype (59.57%), followed by HPV53 (14.79%). Other genotypes were found at lower frequencies. The phylogenetic analysis of HPV isolates showed that the distribution of HPV sequences in Moroccan women with cervical cancer is mainly linked to European, Saudi Arabian and North African epidemiological conditions, suggesting recombinant HPV forms. Additionally, American isolates formed two distinct outgroups, likely due to geographical distance, indicating variations in HPV strains by region.

**Conclusion:**

Morocco faces a significant burden of CC, with HPV being the primary cause. Lifestyle risk factors like low education, male sexual behavior, multiple pregnancies, and sexual intercourse also contribute.

## Introduction

Cervical cancer (CC) is the fourth most common cancer affecting women globally, significantly impacting global health with approximately 604,127 new cases and causing 341,831 deaths annually [1]. It represents a substantial burden within the spectrum of gynecological cancers, distinguished by its high fatality rate [2]. Despite these grim statistics, positive trends have emerged in many countries over the past four decades. There has been a decline in both the incidence and mortality rates of CC, largely due to widespread efforts in screening, early detection, and vaccination campaigns [3]. However, projections suggest a nearly 50% increase in global CC incidence by 2030, which is concerning [4].

The primary origin of CC is attributed to HPV infection, constituting approximately 99.7% of cases [5]. The extensive diversity of HPV is evident through over 200 genotypes, categorized as low or high-risk based on their oncogenic potential [6,7]. The high-risk HPVs (hrHPV) - notably, HPV 16, 18, 31, 33, 34, 35, 39, 45, 51, 52, 56, 58, 59, 66, 68, and 70 - are implicated in the development of various cancers, including cervical, anal, penile, vulval, vaginal, and oropharyngeal cancers [6,7]. HPV 16 and 18, in particular, exhibit heightened oncogenicity, contributing significantly to nearly 50% of high-grade cervical pre-malignancies [8]. However, it is crucial to recognize that HPV infection, while a necessity, does not suffice as the sole cause of CC [9]. Additional factors, such as smoking, immunosuppression, poor sexual health, and screening non-attendance, have been reported to augment the risk of CC [10].

In Morocco, CC emerges as the second most prevalent malignancy among women, second only to breast cancer. Every year, about 10.4 out of 100,000 women are diagnosed with CC, and sadly, 5.8 out of 100,000 women lose their lives to it [1]. Morocco has recognized the urgency of addressing this issue and has committed to the World Health Organization's global initiative to eliminate CC as a public health concern by 2030. With an annual standardized incidence rate of 10.4 per 100,000 women and a mortality rate of 5.8 per 100,000 women, Morocco has outlined a comprehensive National Plan for Cancer Prevention and Control (2020-2029). This plan strategically integrates interventions such as the incorporation of HPV vaccination in the national immunization program for 11-year-old girls, HPV genotyping surveillance, and the implementation of robust screening and management programs for precancerous lesions and CC cases [11,12].

While limited case-control studies have pinpointed HPV as the predominant factor in over 90% of CC cases, methodological constraints, such as the absence of age matching, have been acknowledged. Furthermore, the role of specific viral hosts and various factors, encompassing socio-demographic, behavioral, and genetic elements, in the progression from HPV infection to invasive disease among Moroccan women remains unclear. Nonetheless, a significant knowledge deficit exists regarding the prevalence and distribution of HPV genotypes linked to CC in Morocco. It is imperative to address these informational gaps, as doing so is crucial for refining preventive and therapeutic strategies, ultimately contributing to the ambitious goal of eliminating CC in Morocco [13,14]. Here we conducted a case-control study in Morocco to investigate the risk factors related to CC, including socio-economic status, sexual behavior, reproductive history, contraceptive practices, hygienic conditions, history of sexually transmitted infections, smoking status, and HPV infection and prevalence. In addition, we performed a phylogenetic analysis of HPV sequences to characterize the genetic diversity of HPV types circulating in Moroccan women and explore potential links between viral variants and CC development.

## Methods

**Study design and setting:** this study is a hospital-based case-control study conducted between November 2020 and April 2023 in Rabat and Casablanca, Morocco. It aimed to assess the association between different types of HPV, various risk factors, and the risk of CC. The study utilized a hospital-based design, with cases recruited from the National Institute of Oncology in Rabat and controls selected from Hospital Cheikh Khalifa ibn Zaid in Casablanca. While formal matching criteria were not applied, the study employed multivariate logistic regression analysis to adjust for potential confounding variables, including age, socio-economic status, and sexual behavior. While formal matching criteria were not applied, statistical adjustments were made post hoc to account for these factors in the analytical approach.

**Participants:** this study was conducted between November 2020 and April 2023 and included 169 women with CC and 100 control women. A case was defined as a woman aged 35 to 65 years, diagnosed with CC, and having completed treatment with radiotherapy at the National Institute of Oncology in Rabat. A control was defined as a woman aged 18 to 62 years, with no history of CC and no detectable HPV infection, selected from Hospital Cheikh Khalifa ibn Zaid in Casablanca. The cases were recruited through a consecutive sampling method from the oncology institute, ensuring accurate diagnosis and treatment status. Controls were selected through a convenience sampling approach, where participants were recruited based on their availability and willingness to participate among hospital attendees who met the study's eligibility criteria. Although the control group consisted of generally healthy women attending a private clinic for routine consultations, they may not be fully representative of the general population. Women seeking care in private clinics often have better healthcare access and higher socio-economic status, which may influence their exposure to CC risk factors. This study followed a cumulative case-control design. No specific matching criterion was applied.

**Variables:** the main outcome of the study was the presence of CC. The exposure variable was HPV infection, along with other potential risk factors including socio-economic status, sexual behavior, reproductive history, contraceptive practices, hygiene, history of sexually transmitted infections, and smoking status. Age, socio-economic factors, and sexual behavior were considered as potential confounders. The diagnosis of HPV infection was confirmed by Sanger sequencing, and CC diagnosis was based on clinical and pathological evaluations.

**Data sources/measurement:** data were collected separately for cases (women with CC) and controls (healthy women) using standardized questionnaires, pelvic examinations, and molecular testing methods. For cases, data collection involved a structured questionnaire that was administered through face-to-face interviews conducted by trained healthcare professionals. The questionnaire was originally designed in French and translated into Moroccan Arabic (Darija) to ensure participant comprehension. It was developed by the research team in collaboration with public health and oncology experts to cover key risk factors related to CC. The questionnaire gathered detailed information on socio-economic status, sexual behavior, reproductive history, contraceptive practices, hygiene conditions, history of sexually transmitted infections (STIs), and smoking status. Information regarding husbands´ lifestyles was obtained solely from their wives. In cases where the wife did not have accurate information, the data were considered missing. Clinical examinations, including pelvic examinations, were performed by trained gynecologists to collect exfoliated cervical cells for Pap smear preparation and HPV testing.

**Samples collection and processing:** the samples were collected using a cytobrush for the endocervix, and the collection instruments were rinsed in phosphate-buffered saline (PBS). The cell suspensions were centrifuged at room temperature for 10 minutes at 3,000g, and the cell pellets were stored at -80°C until HPV-DNA detection and typing could be performed. Cases and controls were not selected based on HPV status; however, HPV infection was assessed as a key variable in the study. For HPV-DNA detection and typing, genomic DNA was extracted using the Phenol/Chloroform Extraction method. A conserved region of the HPV-L1 gene was amplified using nested PCR with MY09/MY11 and GP5+/GP6+ consensus primers. Positive PCR products were purified using the PCR purification ExoSaP-IT clean-up system (USB-USA) and directly sequenced using the BigDye® Terminator v3.1 Cycle Sequencing Kit (Applied Biosystems). The nucleotide sequences obtained were aligned and compared with known HPV genotypes in the GenBank database using the online BLAST 2.0 software at National Library of Medicine (NH). The use of consistent sampling methods, standardized molecular techniques, and uniform questionnaires for both cases and controls ensured that the data collected were reliable and allowed for direct comparison between the two groups. For controls, similar data were collected using the same standardized questionnaire to ensure comparability. Controls also underwent pelvic examinations to collect exfoliated cells for Pap smear analysis and HPV testing. The collection and processing procedures, including the use of a cytobrush, PBS rinsing, centrifugation, and storage of cell pellets at -80°C, were identical to those used for cases.

**Bias:** as a case-control study, this research is inherently subject to certain biases, including selection bias and recall bias. Efforts were made to minimize these, but they cannot be entirely eliminated. Selection bias was considered by recruiting cases from a specialized oncology center (National Institute of Oncology, Rabat) and controls from Hospital Cheikh Khalifa ibn Zaid, Casablanca, a private clinic where women attended routine consultations. While controls were generally healthy women with no history of CC or HPV infection, their selection from a hospital-based setting rather than the general community may introduce selection bias, as they may differ in healthcare access and health awareness compared to the broader population. Recall bias remains a concern, as participants may not accurately remember past exposures. While the use of structured, face-to-face interviews ensured consistency in data collection, it does not completely eliminate recall bias, as participants' recollections may still be influenced by their health status or personal perceptions.

**Study size:** the sample size was determined based on the need to detect statistically significant associations between CC and multiple risk factors, including socio-economic status, reproductive and sexual health behaviors, contraceptive use, smoking, and HPV infection. To estimate the required sample size, we used Epi Info™ version 7.2 for case-control studies. The calculation was based on an expected prevalence of key risk factors in Moroccan women, informed by previous epidemiological studies. We assumed that certain major risk factors (e.g., low education level, multiple pregnancies, or HPV infection) would be present in approximately 30-40% of cases and 5-15% of controls. To detect an odds ratio (OR) of at least 3.5 to 4.0-which reflects meaningful epidemiological associations observed in prior studies—while maintaining a 95% confidence level and 80% power, the minimum required sample size was estimated at 160 cases and 90 controls. Given the possibility of missing data or non-responders, we increased recruitment slightly, arriving at a final sample of 169 cases and 100 controls. This sample size provides sufficient statistical power to analyze the relationship between CC and multiple risk factors while allowing for subgroup analyses.

**Quantitative variables:** quantitative variables in this study included age, socio-economic status, sexual behavior, reproductive history, and smoking status. Age was treated as a continuous variable and also categorized into age groups (e.g., 18-30, 31-45, 46-65 years) to facilitate subgroup analyses. Socio-economic status was quantified using a composite score based on factors such as education level, health insurance status, and employment status, allowing for classification into low, middle, and high socio-economic groups. Sexual behavior variables included the age at first intercourse, which was recorded as a continuous variable and categorized into clinically relevant groups (e.g. <18 years, 18-25 years, >25 years). The number of lifetime sexual partners and the husband's number of sexual partners were also collected as numerical variables and analyzed using predefined categories (e.g. 0-1, 2-4, ≥5 partners). Reproductive history variables included the number of pregnancies, parity, and contraceptive use. The number of pregnancies and live births were treated as continuous variables, while contraceptive use was categorized into types of contraception (e.g. hormonal, barrier, none). Smoking status was classified as a categorical variable (current smoker, former smoker, non-smoker) and, when relevant, quantified by the number of cigarettes smoked per day.

**Statistical analysis:** to investigate the risk factors related to CC, including socio-economic status, sexual behavior, reproductive history, contraceptive practices, hygienic conditions, history of sexually transmitted infections, smoking status, and HPV infection and prevalence, we conducted statistical analyses to assess their associations with CC. We used unconditional logistic regression to calculate odds ratios (ORs) with 95% confidence intervals (CIs), quantifying the strength of associations between each risk factor and CC. To further refine these findings, we performed multivariate conditional logistic regression with a reduction strategy to control for potential confounders and determine which factors remained independently associated with CC. All variables that showed statistical significance in the bivariate analysis (P < 0.05), except for HPV and residence area, were included in the multivariate model. Additionally, stratified analyses were applied to explore potential interactions between risk factors, particularly how HPV infection, sexual behavior, reproductive history, and socio-economic factors may influence CC risk. These analyses allowed us to distinguish between independent risk factors and those acting in combination, providing a clearer understanding of their individual and joint contributions to CC development. Through this analytical approach, we were able to answer our research question by identifying the key risk factors for CC in Moroccan women and evaluating their impact, both independently and in interaction with other variables. This enabled us to determine the most relevant factors contributing to CC risk, informing potential preventive strategies and public health interventions. All statistical analyses were performed using SPSS version 29.0.10, GraphPad Prism version 8.0.2, and Microsoft Office Excel.

**Phylogenetic tree generation:** we generated a phylogenetic tree of HPV using nucleotide sequences obtained in this study. Reference sequences for 12 HPV lineages were collected from the GenBank database. The sequences were aligned using the MAFFT tool to produce a multiple sequence alignment (MSA). We then constructed the phylogenetic tree using the RAxML tool, providing insights into the genetic relationships of the HPV types identified in this study.

## Results

A total of 269 women were enrolled in the study between 2020 and 2023, including 169 women diagnosed with CC and 100 control women. All cases received radiotherapy and chemotherapy at the National Institute of Oncology (INO) in Rabat, while controls were recruited from Hospital Cheikh Khalifa ibn Zaid in Casablanca, Morocco. Among the 169 cases, 157 were included in the final analysis, with 12 women (7.10%) excluded due to missing International Federation of Gynecologists and Obstetricians (FIGO) classification data. The control group maintained full participation throughout the study, with no reported dropouts. The main reasons for non-participation among cases included incomplete data (12 participants) and advanced metastatic disease or inability to undergo required treatments. The average age of women with CC was 49.75 ± 0.95 years, and all 169 cases were diagnosed with squamous cell carcinoma. According to FIGO staging, most cases were classified as Class II, with fewer in Classes I and III. The study collected data on socio-demographic, reproductive, and sexual behavior factors for both cases and controls. Control women had an average age of 49.2 ± 0.98 years and generally exhibited more favorable socio-economic and hygienic conditions compared to cases. The control group also reported fewer pregnancies and a lower prevalence of smoking. The study had minimal missing data, primarily related to FIGO classification, which led to the exclusion of 12 cases. Other variables, including socio-economic status, reproductive history, and sexual behavior, had less than 5% missing data, which were managed through listwise deletion during statistical analysis.

### Risk factors to CC in Morocco

***Socio-demographic and socio-economic risk factors:*** the study found that both the cases and controls had similar average ages, with values of 49.75 ± 0.95 and 49.2 ± 0.98, respectively (p < 0.001). All 169 cases were identified as squamous cell carcinoma, and there were no instances of adeno/adenosquamous carcinoma. The bivariate analysis (Table 1) examined the associations between socio-demographic factors, including age, marital status, health insurance, residence area, education, socio-economic level, employment outside the home, and the risk of CC. The absence of health insurance significantly increased the risk of CC by 17.80 times. Separated or divorced women had a 14.444 times higher risk compared to married women. Women without education had a higher risk, with an odds ratio (OR) of 9.167 compared to educated women. Additionally, those with low socio-economic status faced a much higher risk (OR = 5.592) than those with middle or high socio-economic status. Living in rural areas was linked to a 4.755 times greater risk of CC. The multivariate analysis (Table 1) revealed that women without formal education had a higher chance of developing cancer (OR vs. educated = 4.5). The risk of CC is significantly raised by not having health insurance, increasing it by 3.7 times.

**Table 1:** demographic and socio-economic factors associated with cervical cancer risk among 169 cases and 100 controls in Morocco (November 2020 - April 2023): a comparative analysis

	Cases		Controls		ORa (95% CI)	P value	ORb (95% CI)	P value
	No	(%)	No	(%)				
**Marital status**								
Single	1	0,60	5	5	1		1	
Married	96	58,53	78	78	14,4(1,4-140,78)	0,22	2,01(0,6–3,0)	NS
Widowed	41	25	8	8	2,3(1,039-5,302)	0,40	1,40 (0,5–3,9)	NS
Separated-divorced	26	15,58	9	9	0,5(0,193-1,646)	0,294	-----	
**Health insurance**								
Yes	139	84,75	99	99	1		1	
No	25	35,36	1	1	17,8(2,37-33,60)	0,05	3,1 (2,1–6,5)	0.046
**Residence area**								
Urban and sub-urban	106	64,63	90	90	1		-----	
Rural	58	35,36	10	10	4,7(2,293-9,858)	0,01	------	NS
**Education**								
High	9	5,48	65	65	1		1	
Primary	34	20,73	25	25	87,3(33,8-225,8)	0,001	4,5 (2,1–12,0)	0.05
Illiterate	121	73,78	10	10	9,1(4,004-20,98)	0,001	----	0.013
**Socio-economic level**								
Middle and high	109	66,46	92	92	1			
Low	55	33,53	8	8	5,5(2,529-12,36)	0,001	2,9 (1,8–4,2)	0.473
**Work outside home**								
Never	139	84,75	50	50	1			
Yes	25	15,24	50	50	0,16(0,092-0,29)	0,001	1,7 (1,2–3,8)	0.293

ORa, odds ratio of the bivariate analyses; ORb, odds ratio multivariate analyses; CI, confidence interval;1 Reference category. NS: Not Significant.

***Reproductive factors and contraceptive methods:*** in the present study, women who experienced menarche at ages 13 to 14 had a 6.8-increased chance of having CC compared to those who had menarche at 15 or older. Women who reached menopause between 45 and 49 years, compared to those who reached menopause at age 50 or older, also faced higher risks, with ORs of 4.433 and 2.111, respectively. The number of pregnancies was an important factor, as women with four or more pregnancies had a higher risk than those with three or fewer pregnancies (OR = 3.971). Having the last pregnancy after age 35 was associated with a higher risk of CC (OR = 3.971). Women who had their first pregnancy between ages 19 and 22, had an 0.123 increased risk compared to those who had their first pregnancy at 18 or younger. However, the analysis did not find any significant associations between the duration of oral contraceptive use, the utilization of injectable contraceptives, condom use, and CC risk (Table 2). Multiple substantial risks for developing CC were identified by the multivariate analysis of reproductive variables. Women who began menstruating between the ages of 13 and 14 were six times more likely to develop CC. Having the last pregnancy before age 35 tripled the risk. Getting into menopause between ages 45 and 49 increased the odds by 4.433 times. Additionally, women who started having sex before age 18 faced a notable risk (OR = 0.012).

**Table 2:** reproductive factors, contraceptive methods, and screening variables among 169 cases of cervical cancer and 100 controls in Morocco (November 2020 - April 2023): a comparative analysis

	Cases	Controls			ORa (95% CI)	P value	ORb (95% CI)	P value
	No.	(%)	No.	(%)				
**Age at menarche**								
≤ 12	39	23,78	51	51	1		1	
13-14	77	46,95	40	40	6,80(2,984-15,494)	0,11	6,4 (2,5-7,9)	0.002
≥ 15	48	29,26	9	9	2,84(1,267-6,386)	0,001	3,6 (1,7-5,4)	0.001
**No of pregnancies**								
≤ 3	51	31,09	79	79	1		1	
> 3	113	68,90	21	21	9,10(5,028-16,502)	0,001	0,7 (0,1-2,0)	NS
**Age at first pregnancy**								
≤ 18	81	53,64	10	10	1		1	
19-22	43	28,47	43	43	0,12(0,053-0,288)	0,001	1,2 (1,1-3,4)	NS
> 22	27	17,88	27	27	1(0,506-1,975)	1	0,5 (0,2-1,0)	
**Age at last pregnancy**								
≤ 35	83	58,54	8	8	1		1	
> 35	59	41,54	11	11	3,971(1,923-8,200)	0,001	0,7 (0,5-1,3)	0.001
**Menopause**								
No	16	9,75	65	65	1		1	
Yes	148	90,24	35	35	13,08(7,079-24,200)	0,001	1,05(0,7-3,2)	0.004
**Age at menopause**								
≥ 50	44	29,72	21	21	1			
45-49	26	17,56	6	6	4,43(1,814-10,838)	0,001	2,2 (0,8-6,5)	0.001
< 45	78	52,70	8	8	2,11(0,671-6,640)	0,201	1,1 (0,8-5,9)	NS
**Years of use of oral contraceptives**								
< 6	11	40,74	7	7	1		1	
≥ 6	16	59,25	23	23	0,69(0,263-1,810)	0,452	0,9 (1,1-2,1)	NS
**Use of injectable contraceptives**								
No	152	92,68	98	98	1		1	
Yes	12	7,31	2	2	3,01(0,348-26,211)	0,316	0,7 (0,3–2,0)	NS
**Condom use**								
No	163	99,39	100	100	1			
Yes	1	0,60	0	0	----------	0,999	1,2 (1,4–5,5)	NS

ORa, odds ratio of the bivariate analyses; ORb, odds ratio multivariate analyses; CI, confidence interval;1 Reference category. NS: Not significant.

***Sexual behavior:*** in the analysis presented in Table 3, women who never or only occasionally washed the genital area after intercourse showed a significantly higher risk of CC, with 27.871 times increase, compared to those who consistently maintained genital hygiene. A history of sexually transmitted infections was significantly associated with elevated risks of CC, with ORs of 2.173, 4.248, and 14.370, respectively. Women whose husbands had two or more sexual partners exhibited a heightened risk (OR = 6.305) compared to those with husbands who had only one partner. With an OR of 2.351, women who had intercourse while menstruating were more likely to experience CC due to poor hygiene habits. Additionally, the number of sexual partners husbands had played a significant role in starting sexual activity before the age of 18 emerged as a noteworthy risk, with an odds ratio (OR) of 0.121 compared to those starting at 18 years or older. Moreover, there was no discernible association found between the number of lifetime sexual partners and CC risk. According to multivariate analysis, women whose husbands had two or more sexual partners saw a six-time rise in CC compared to those whose husbands had only one relationship. Additionally, women with a history of sexually transmitted infections faced a significantly higher risk (OR = 2.173) (Table 3).

**Table 3:** women's sexual characteristics, sexually transmitted infections, and hygienic practices among 169 cases of cervical cancer and 100 controls in Morocco (November 2020 - April 2023): a comparative study

	Cases	Controls			ORa (95% CI)	P value	ORb (95% CI)	P value
	No.	(%)	No.	(%)				
**Age at first intercourse**								
≥ 18	41	25,78	72	74,2	1		1	
< 18	118	74,21	25	25,7	0,121(0,068-0,21)	0,001	1,2 (1,8-2,9)	0.001
**No of lifetime sexual partners**								
1	127	80,37	97	100	1			
≥ 2	31	19,62	0	---		0,998	0,7 (0,2-2,3)	NS
**Sexual intercourse during menstruation**								
No	107	65,63	80	80	1		1	
Yes	57	28,65	20	20	2,351(1,327-4,16)	0,003	2,3 (1,3–5,7)	NS
**Husband’s number of sexual partners**								
One	30	52,63	90	92,7	1		1	
Two and more	27	47,36	7	7,21	6,305(2,725-14,5)	0,001	6,7(3,3–8,2)	0.001
**History of sexually transmitted infections**								
No	98	59,75	75	75	1		1	
Yes	66	40,24	25	25	2,173(1,259-3,75)	0,005	16,7(11,9–23,6)	0.003
**History of herpes infection**								
No	122	74,39	97	97	1		1	
Yes	42	25,60	3	3	4,248(1,986-9,08)	0,001	0,3 (0,1–1,1)	NS
**History of condyloma infection**								
No	121	73,87	96	96	1		1	
Yes	43	26,21	4	4	14,37(4,35-47,44)	0,001	1,3 (0,7–3,2)	NS
**Genital washing after intercourse**								
Always	121	77,5	96	96	1		1	
Sometimes or never	43	22,5	4	4	27,8(3,75-206,92)	0,001	1,8 (1,1–3,7)	NS

ORa, odds ratio of the bivariate analyses; ORb, odds ratio multivariate analyses; CI, confidence interval;1 Reference category. NS: Not Significant.

***HPV infection:*** the objective of this analysis is to investigate the various HPV genotypes associated with CC in a cohort of 169 women who received radiotherapy and chemotherapy, alongside 100 control women. The results of this analysis revealed a detection rate of 33.81% among all cases, encompassing 82.24% of squamous cell carcinomas. In contrast, only 8% of control women displayed evidence of HPV infection. It's worth noting that the prevalence of HPV among cases may be influenced by ongoing radiotherapy treatments (Table 4). The analysis identified eight different HPV genotypes, with HPV 16 being the predominant type among both cases (59,57%) and control women (75%), followed by HPV 53 and 18. Women with any HPV infection had markedly elevated odds of CC, displaying an odds ratio (OR) of 4,430 (95% CI: 1,997-9,830) compared to those without HPV. Moreover, specific genotypes demonstrated varying degrees of association with CC, with an OR of 48.3 (95% CI: 18.2-408.0) for HPV 16 infection and 30.8 (95% CI: 2.1-402.0) for HPV 53 infection, highlighting the differing risks associated with distinct HPV genotypes (Table 5).

**Table 4:** distribution of HPV infections and HPV genotypes among 169 cases of cervical cancer and 100 controls in Morocco (November 2020 - April 2023)

	Total cases		Squamous cell carcinoma		Adenocarcinoma		Controls	
	No.	(%)	No.	(%)	No.	(%)	No.	(%)
**Total HPV tested**	139	82.24	139	82.24	0	0	100	100
**HPV Negative**	92	66.18	92	54.43			92	92
**HPV Positive**	47	33.81	47	27.81			8	8
**High-risk types**	33	70.21	33	19.52			8	100
**Low-risk types**	10	21.27	10	5.91			0	0
**HPV types**								
16	28	59.57	28	59.57			6	75
18	3	6.38	3	6.38			1	12.5
53	7	14.79	7	14.79				
83	1	2.12	1	2.12				
31	1	2.12	1	2.12				
89	1	2.12	1	2.12				
66	1	2.12	1	2.12				
62	1	2.12	1	2.12				
**Not identified**	4	8.51	4	8.51			1	12.5

High-risk types (HPV 16, 18, 31 and 66). Low-risk HPV: (53,83,89,62), Not identified: HPV genotypes were not identified in the NCBI database.

**Table 5:** comparative analysis of HPV infection among 169 cases of cervical cancer and 100 controls in Morocco (November 2020 - April 2023)

	Cases		Controls		p value	OR (95% CI)
	No.	(%)	No.	(%)		
**HPV genotyping**	139	100	100	100		
**Negative**	92	66.18	92	92	1	
**Positive**	47	33,81	8	100	0,001	4,43(1,99-9,83)
**HPV number of genotypes**	8		2			
**Genotypes of HPV**						
16	28	65,11	6	75	0,001	48.3(18.2–408.0)
18	3	6,97	1	12.5	0,4	29.6 (2.2–307.0)
53,83,89,62	10	23,25			0,001	28.8(1.1–302.0)
31,66	2	4,65			5,0	0.6 (0.1–4.9)

OR, odds ratio; CI, confidence interval;1 Reference category, HPV, human papillomavirus, Not identified: HPV genotypes were not identified in the NCBI database.

***Association between HPV Infection and the significant associated risk factors:*** Table 6 shows an interesting association between HPV infection and a higher number of pregnancies, particularly those exceeding three. Factors such as education level, marital status, having had five or more births, experiencing menopause at the age of 45 years, and a woman's report of her husband's extramarital sexual relationships were not found to be associated with HPV infection.

**Table 6:** association between HPV infection and significant risk factors among all women in Morocco (November 2020 - April 2023): a comparative analysis

	p value	OR (95% CI)
**Education**		
High		1
Primary	0.108	3.761(0.225-1.384)
Illiterate	0.722	1.221(0.298-1.567)
**Age at first intercourse**		
≥ 18		1
< 18	0.663	1.228(0.629-2.733)
**No of pregnancies**		
≤ 3		1
> 3	0.002	0.358(0.185-0.692)
**History of sexually transmitted infections**		
No		1
Yes	0.846	1.067(0.611-2.316)
**History of herpes infection**		
No		1
Yes	0.974	1.017(0.478-2.645)
**History of condyloma infection**		
No		1
Yes	0.658	1.298(0.323-1.811)
**Genital washing after intercourse**		
Always		1
Sometimes or never	0.123	2.493(0.629-4.278)

OR, odds ratio; CI, confidence interval;1 Reference category.

**Figure 1:**
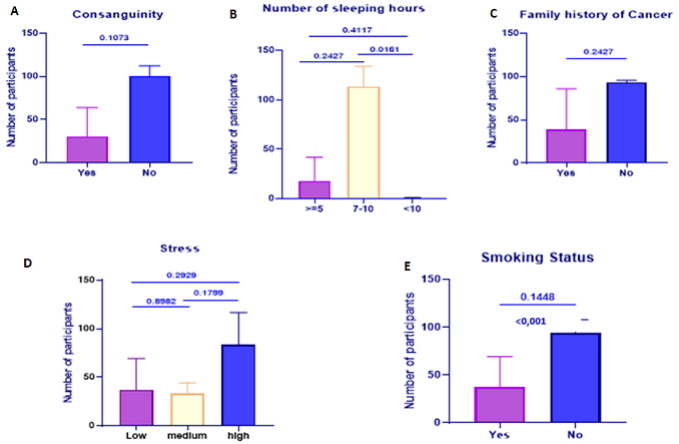
comparative analysis of the associations between cervical cancer and various risk factors in Morocco (November 2020 - April 2023): A) consanguinity; B) sleeping hours; C) family history of Cancer; D) stress; E) smoking status

***Other factors:*** in the present study, 36.58% of cases versus 15% of controls had a history of smoking. Statistical analysis showed that smoking was significantly associated with the risk of developing CC, raising it 4-fold. In contrast, our analysis revealed no statistically significant association between CC and factors such as family history of cancer, consanguinity, number of hours of sleep, and stress (Figure 1).

**Figure 2:**
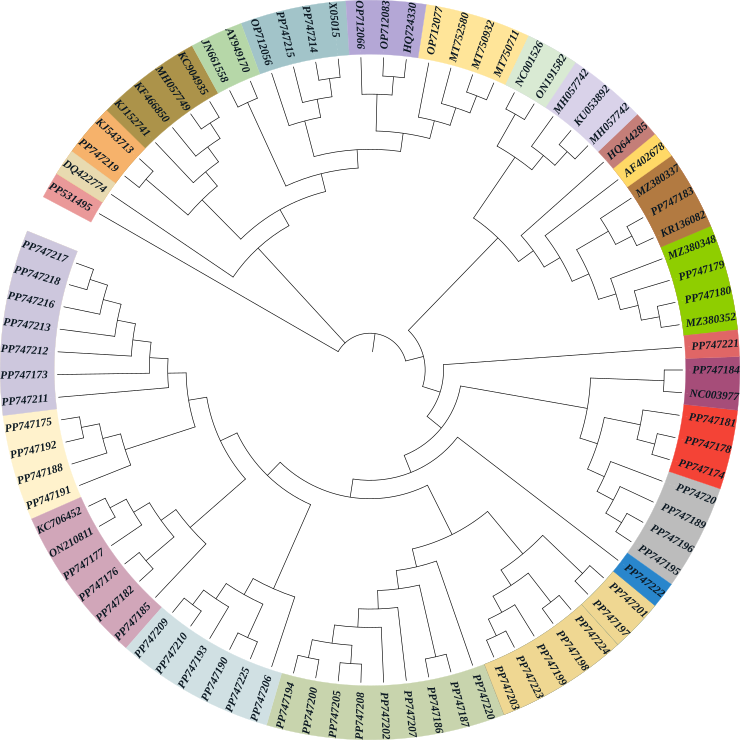
neighbor-joining phylogenetic tree of HPV strains identified in Morocco compared to global HPV strains (November 2020 - April 2023)

**Phylogenetic analysis:** regarding exploring the landscape of Moroccan HPV genotypes associated with CC, we utilized the phylogenetic tree approach. A total of 139 sequences were successfully sequenced, with 47 positively identified genotypes. Five sequences remained unidentified upon comparison with the NCBI database. All sequenced data were curated and have been published on GenBank, with corresponding accession numbers. To enhance the valorization of our findings, we extracted 33 HPV sequences published on the NCBI platform, representing different genotypes from various countries, including HPV16 (NC001526, KU053892, HQ644285, NC03977, AF402678, KJ152741, KC904935, KF466850, MH057742, OP712015, DQ422774, MH937413, LC456183, ON191582 from the USA, Costa Rica, Europe, Asian-American, Spain, Morocco, Algeria, Saudi, France, Turkey, Italy, Japan, and Netherlands), HPV18 (X05015, KJ543713, OP712056, MH057749 from Spain, France, and Saudi), HPV31 (MT750932, ON210811, MT752580, MT750711, KC706452 from Algeria, Tunisia, Spain, Morocco, and Saudi), HPV53 (MZ380337 from Tunisia), HPV62 (MZ380348, OP712066 from Tunisia and France), HPV66 (AY949170, OP712077, JN661558, HE798639 from Morocco, France, Italy, and Kuwait), HPV83 (HQ724330 from France), and HPV89 (MZ380352, OP712083 from Tunisia and France) respectively. In total, 86 patterns and 12 lineages of sequences participated in constructing this phylogenetic tree (Figure 2).

The AF402678AMR and NC001526HPV isolates represent HPV16, both utilized as reference points from the USA, forming two distinct outgroups within the analysis, signifying their lack of association with the examined isolates under study. Isolate PP531495 is the most ancestral among the studied isolates, sharing a closer genetic relationship with their common ancestors. KC706452.1 (HPV16) from Saudi Arabia, and JN661558 (HPV66) from Italy, exhibit a common lineage with PP747215, PP747214, and PP747219 isolates from our population. Advancing to lineages with reference isolates, the Algerian isolate MT750932ALG (HPV31) and Moroccan isolate PP747184, within the same lineage, acted as progenitors for six additional lineages. The Tunisian isolate MZ380337.1 associated with HPV53 underwent 11 mutations and serves as the source for two Moroccan isolates, PP747179 and PP747180. Isolates PP747185, PP747182, and PP747191 share a common ancestral lineage with the reference isolate ON210811UN (HPV31) from Tunisia. The phylogenetic tree reveals that Moroccan samples mostly share ancestry with North African, Saudi Arabian, and European strains within specific lineages. In contrast, American isolates form two distinct outgroups due to geographical separation. The presence of European and North African isolates in Moroccan lineages is likely due to immigration, geographical proximity, and shared cultural ties among North African countries.

## Discussion

This study explores risk factors for CC and HPV infection prevalence in Morocco among 169 cases and 100 controls, highlighting global efforts to manage CC progression. In Morocco, delays in diagnosis are a challenge. Our study found that 53.23% of patients were diagnosed with SCC at stage II, aligning with previous reports [15,16]. In this study, the lack of health insurance significantly contributes to CC development in Moroccan women due to limited access to diagnosis and screening. This aligns with other studies showing health insurance as a major risk factor for CC, with an odds ratio (OR) of 3.1 [13]. Many studies have explored the link between education and the risk of developing CC, worldwide [13,17,18]. Our findings indicate that Moroccan women with low education levels are nine times more likely to develop CC compared to those with higher education levels. Socio-economic status is a known risk factor for CC. Our study confirms that low socio-economic backgrounds are significantly correlated with CC incidence (p < 0.001), consistent with national and international findings on the impact of education [13,19,20].

In our study, women in rural areas often have poor hygiene, multiple deliveries with inadequate follow-up, and limited incomes. They also face restricted healthcare access, poor nutrition, and low health awareness, all contributing to a higher risk of CC [21,22]. Various reproductive factors and contraceptive methods influence the risk of developing CC. Our research shows that Moroccan women who begin menstruating at age 13 or older face a higher risk of CC compared to those who start before age 13. This finding aligns with other Moroccan studies but contrasts with Chinese studies, which found no such association [21]. This study finds that multiparous women, those with multiple pregnancies, have an increased risk of CC. Statistical analysis shows a significant association between early-age pregnancies and CC risk (p-value = 0.001), consistent with other studies. In this study, 72% of Moroccan CC patients were multiparous (four or more pregnancies), 24% had one to three children, and 4% were nulliparous. Similar trends were observed in Costa Rica [23], but this was not reported in Denmark, the US, Colombia, or Spain [24].

In a pooled analysis of 10 IARC case-control studies, women with 5-6 or 7+ full-term pregnancies had odds ratios of 5.0 and 8.3, respectively, compared to nulliparous women [25]. A weaker association with parity was observed in developed countries, where the parity range studied was smaller [26,27]. Several studies in Morocco and worldwide confirm the association between menopause age and CC. Our research supports this with a p-value less than 0.001, highlighting the significant link between menopause and CC in both Moroccan and global contexts. Most CC patients in this study never used oral contraceptives, consistent with findings from Colombia, Spain, Denmark, and the US, showing no link between oral contraceptive use and pre-malignant lesions in HPV-positive women [28]. However, several studies suggest that using hormonal contraceptives for over five years may increase the risk of CC in HPV-positive women. The adjusted relative risk is 2.82 for 5-9 years of use and 4.03 for 10+ years [29]. This study found a strong correlation (p-value <0.05) between early age at first marriage and CC. Women with their first sexual experience before age sixteen are twice as likely to develop CC compared to those after age twenty, likely due to the cervix's increased susceptibility to carcinogens during adolescence [30,31].

Contrary to global findings, the number of sexual partners did not influence CC development in our study. Most women reported only one sexual partner, with 80.37% of cases and 100% of controls being monogamous. However, 47.36% of the cases and a significant portion of the controls' partners engaged in polygamy, indicating this practice is more common among men [24]. Poor hygienic conditions can lead to genital infections and chronic CC. Previous studies show that women who maintain better hygiene, such as washing the genital area during menstruation, have a reduced risk of developing CC [32]. Our study shows a significant link between husbands' sexual behavior and CC incidence in Morocco. This finding aligns with previous research, highlighting the importance of considering husbands' sexual behavior as a key factor in CC development both nationally and globally [13,21,33].

Our study identifies a history of sexually transmitted infections (STIs) as a risk factor for CC in Moroccan women. This finding aligns with other studies consistently showing that STI exposure is associated with CC [34,35]. Our study is the first in the Moroccan population to find a significant association between smoking and CC (p < 0.001). Among Moroccan women, 61.58% were active or passive smokers. Studies suggest smoking increases CC risk, especially long-term. Tobacco components like nicotine and cotinine are found in cervical mucus, even in non-smokers exposed to passive smoking. However, the exact biological mechanisms linking smoking to CC are not fully understood [23,28,36].

Numerous global studies and our research find no correlation between CC and factors such as working outside the house, marital status, stress levels, family history of cancer, and sleep duration. These factors appear to have little effect on the risk of CC [13,21,37]. Our study aimed to shed light on the prevalence and genotypes of HPV in Moroccan women, addressing the data gap on the disease, especially in areas with limited healthcare access. All HPV infections identified in CC cases were high-risk types, consistent with findings from global [9] and Moroccan studies [32]. The estimated HPV prevalence of 33.81% in Morocco provides significant insight into the infection landscape. This finding aligns with previous studies in the Fes region and Northern Morocco, which reported prevalence rates of 42% to 45% [30,38]. HPV 16 was the most prevalent type, found in 65.11% of CC cases. This suggests that the distribution of HPV subtypes in Morocco is more similar to Europe and North America than to other parts of Africa, where HPV 16 accounts for less than 50% of cases [31,39].

In line with reports from lower-middle-income countries [40,41], our study highlights that high-risk HPV strains account for a substantial 70.19% of all HPV-positive cases in Morocco. Furthermore, we confirm that HPV16, 18, 31, and 53 are the most prevalent high-risk HPV genotypes, consistent with previous studies in the region [16,32,38]. Our study highlights the critical need for HPV detection and screening, particularly in asymptomatic women. With an estimated 8% prevalence of cervical lesions, early detection and intervention are crucial to reduce CC burden in Morocco. These findings support the implementation of widespread screening programs and policies tailored to regional HPV prevalence variations [40]. We observed regional variations in HPV prevalence, with the central region showing lower rates of 6% to 20%. This geographical variation highlights the need for precise assessments of regional HPV genotypes. Such information is crucial for developing targeted CC prevention strategies, public health initiatives, and policies tailored to the unique epidemiological dynamics in different regions of Morocco [16,32].

The phylogenetic analysis of 60 patterns and 9 lineages revealed that USA isolates were genetically distinct. Isolate PP531495 was the most ancestral, closely related to common ancestors. A single mutation event caused divergence, with Algerian and Moroccan isolates serving as progenitors for multiple lineages. European isolate NC-003977PB influenced two Moroccan isolates, which shared ancestry with North African and European strains. American isolates were separate, reflecting geographical distance. The presence of European and North African isolates in Moroccan lineages suggests that immigration, proximity, and shared culture impact HPV transmission dynamics and genetic evolution. We observed regional variations in HPV prevalence, with the central region showing lower rates of 6% to 20%. This highlights the need for precise regional assessments of HPV genotypes to develop targeted CC prevention strategies, public health initiatives, and policies tailored to the specific epidemiological dynamics of different Moroccan regions [16,32]. This idea suggests that over hundreds of thousands of years, the interactions between the virus and its host have led to some adaptation of the virus to the host. This adaptation is particularly evident in the increased association with cancer development for a common genotype across different demographic groups [42].

**Limitations:** our study has several limitations that should be considered. First, the sampling period coincided with the COVID-19 pandemic, which posed challenges in obtaining the necessary authorizations for sampling and conducting surveys. Second, we were unable to access biopsy samples, limiting the scope of our molecular and histopathological analyses. Additionally, some women declined participation, which may have introduced selection bias. Despite these limitations, our findings provide valuable insights into CC and HPV in Moroccan women and highlight important directions for future research.

## Conclusion

In conclusion, this study highlights the strong association between HPV infection and CC in Morocco. Lifestyle risk factors, such as low education, male sexual behavior, multiple pregnancies, intercourse during menstruation, and a history of STIs, were found to be significantly associated with an increased risk of CC. Therefore, targeted health education and prioritized screening services are essential to address these specific risk factors and effectively combat CC in the Moroccan population.

### What is known about this topic

- CC is one of the most common malignancies among women in Morocco;
- Limited case-control studies have pinpointed HPV as the predominant factor in over 90% of CC cases.

### What this study adds

- Significant associations (P<0.05) were found between CC and educational level (OR=9.167), sexual activity during menstruation (OR=2.351), previous occurrences of sexually transmitted infections (OR=2.173), history of multiple sexual partners by the husband (OR=6.305);
- HPV infection was identified in 33.81% of cases and 8% of controls; the most prevalent genotype was HPV16 (59.57% of infections), followed by HPV53 (14.79%); other genotypes (HPV18, HPV83, HPV31, HPV89, and HPV66) were detected at lower frequencies, each constituting between 2.12% and 6.38% of cases;
- The distribution of HPV sequences in women with CC in Morocco is mainly related to European, Saudi Arabian, and North African epidemiological conditions, indicating the presence of recombinant HPV forms.
